# A life threatening uterine inversion and massive post partum hemorrhage caused by placenta accrete during Caesarean section in a primigravida: a case report

**DOI:** 10.1186/1757-1626-2-138

**Published:** 2009-02-12

**Authors:** Dimitris Tsivos, Fozia Malik, Kirana Arambage, Philip Hagan, Cheng Lee

**Affiliations:** 1 Department of Obstetrics and Gynecology, Southend General Hospital NHS Trust, Prittlewell Chase, Westcliff on Sea, Essex, SS0 0RY, UK

## Abstract

**Background:**

A 32-year-old Caucasian primigravida was admitted for elective Caesarean Section at 36 weeks and 6 days with the diagnosis of preeclampsia.

**Case presentation:**

Traction of the umbilical cord after delivery of a healthy baby resulted in uterine inversion. The placenta was found to be densely adherent to the posterior uterine wall. Piecemeal excision of the placenta as close as possible to the uterine lining was then performed.

**Conclusion:**

In this way we were able to control a massive post partum hemorrhage and preserve the fertility of the patient.

## Background

**Figure 1:**
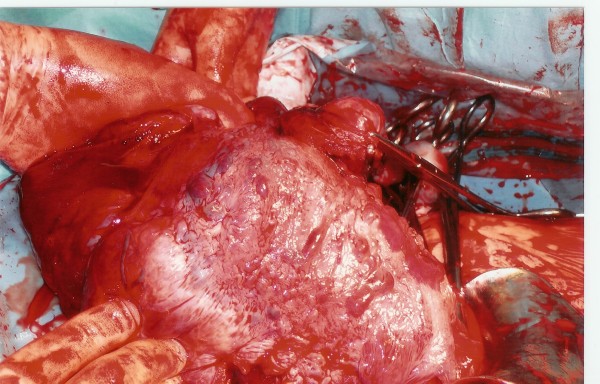
**Uterine inversion secondary to placenta accreta during CS**.

Placenta accreta is defined as abnormal adherence, either in whole or in part of the afterbirth to the underlying uterine wall. Placenta accreta and other pathological placentations (such as increta, percreta) are rare complications of pregnancy with potential life threatening and fertility threatening consequences. The incidence of placenta accreta has increased ten times over the last fifteen years, which reflects the increase in the rate of Caesarean Sections (CS) [1]. Placenta accreta has become the most important cause of peripartum hysterectomy. A life threatening acute uterine inversion and massive PPH can be caused by placenta accreta during CS but seldom in a primigravida (Figure 1).

## Case presentation

A 32 year old, primigravida, was admitted in a District General Hospital for elective Caesarean section at 36 weeks and 6 days with the diagnosis of preeclampsia. She had two antenatal ultrasound examinations showing a healthy fetus and posterior fundal placenta.

The patient had lower segment CS of a healthy male infant under spinal anesthesia. The placenta was found to be densely adherent to the posterior uterine wall. Traction of the umbilical cord was applied and subsequently resulted in uterine inversion.

The placenta was removed by 'piecemeal' excising as close as possible to the uterine lining. About 80% of the placental tissue was removed until the uterine inversion was corrected. The uterus was closed in two layers. Two intra-abdominal drains were sited. The estimated blood loss was 2.5 litres and five units of blood were transfused together with 2 units of FFP during intra-operative and post-operative period. In addition, the patient was treated with intravenous oxytocin infusion, pr misoprostol and antibiotics.

**Figure 2:**
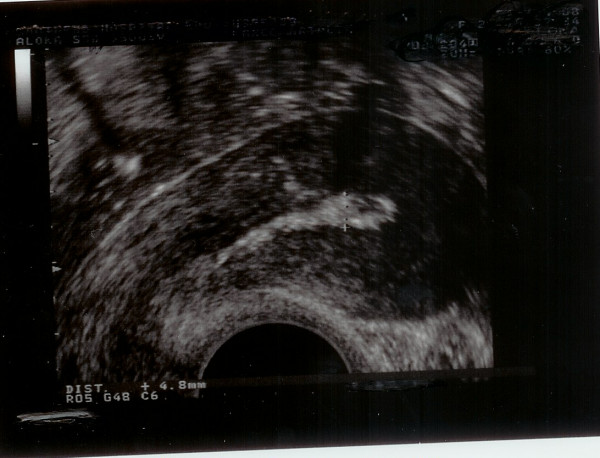
**Retained placental products 2 days post CS**.

On the second post partum day, vaginal Doppler ultrasound scan showed significant amount of placental tissues with increased vascularity measuring 2.7 × 6.6 × 6.8 cms within the endometrial cavity (Figure 2).

**Figure 3:**
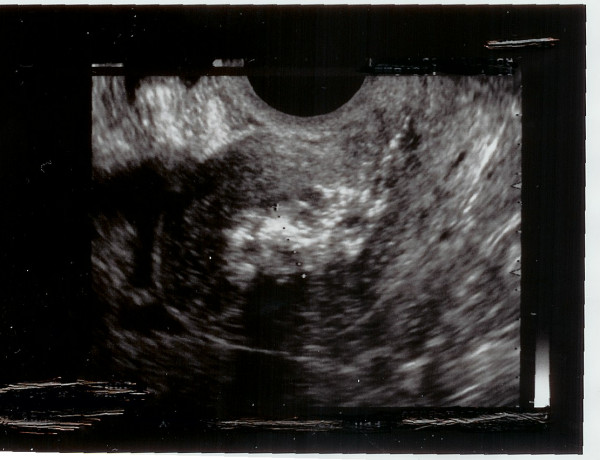
**Retained placental product 2 weeks post CS**.

The patient was discharged on the fifth post-operative day with a conservative management. A follow up Ultrasound scan after two weeks showed reduction of placental mass (Figure 3). In addition, there was significant decrease in serum beta HCG levels from 2300 u/L on day 1 to 13 u/L at four weeks post operatively. The patient remained with minimal vaginal bleeding without abdominal pain. She had two normal periods after stopping breastfeeding and was feeling well. She was discharged from the early pregnancy unit.

## Discussion

A life-threatening uterine inversion can be rarely caused by placenta accreta [2]. Placenta accreta classically presents with retained placenta and hemorrhage. The association between uterine inversion and placenta accreta is unclear, however, strong traction on the umbilical cord with fundal placenta, excessive fundal pressure, relaxed uterus, short umbilical cord, uterine anomalies and antepartum use of magnesium sulphate are known associated factors [2]. Uterine inversion and retain placenta accreta can both be fatal complications [3].

In the case described the placenta accreta was complicated by uterine inversion and subsequent massive post partum hemorrhage, significantly increasing the risk of maternal mortality. Massive post partum hemorrhage is a major cause of maternal mortality in the United Kingdom (why women die latest report) [4].

**Figure 4:**
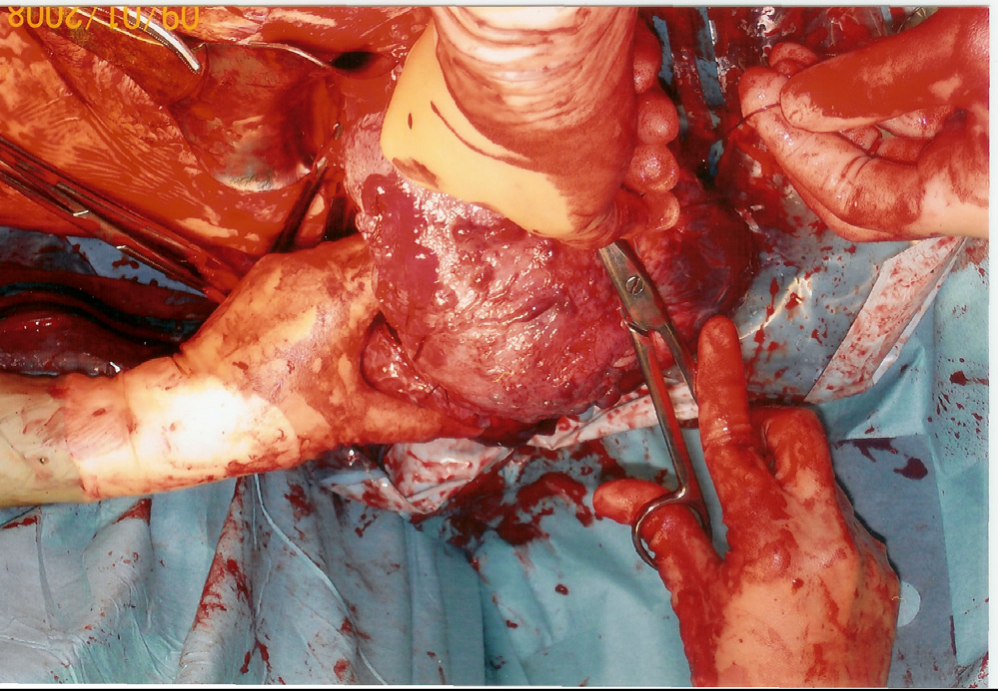
**Correction of uterine inversion following piecemeal excision**.

In this case, the placenta was clamped as close to the uterine cavity as possible and cut. The base of the placenta was overrun with haemostatic sutures and this was repeated until as much of the placenta as possible was removed (Figure 4). Placental removal enabled correction of uterine inversion.

Despite the many conditions associated with uterine inversion risk assessment is often lacking making the condition usually unexpected at the time of presentation. The association between abnormal placentation such as placenta accreta and uterine inversion is well supported [2]. Therefore, we advocate antenatal evaluation and risk assessment for placenta accreta [5]. Prenatal Ultrasound reported sensitivity of 94% and specificity of 79% for placenta accreta, but offer no more than provisional diagnostic probability statement [6]. Moreover, because 45% of placenta accreta cases were not detected by ultrasound, it is important to consider avoiding manual removal of placenta if there were intraoperative signs of accreta [6]. If clinically or sonographically the patient is suspected antenatally to be at risk of placenta accreta, appropriate management options should be considered, such as attempted conservative management or hysterectomy and counseling provided about potential sequelae [6]. The traditional management is abdominal hysterectomy, but this operation terminates fertility and may have devastating psychological effects. However, in correct circumstances, a conservative approach may be suitable. Conservative management of abnormally invasive placentation can be effective and fertility can be preserved. It should be only considered in highly selected cases when blood loss is minimal and there is wish for fertility preservation [7].

For women who want to preserve their fertility the placenta should be left intact if possible after caesarean delivery as this approach lowers the risk of subsequent hysterectomy from 85% to 15%. For women who have completed their family, hysterectomy with placenta left in situ is preferable to lower the maternal morbidity rates [6].

This case report involved conservative management. Peripartum hysterectomy was avoided and the aim was to preserve fertility. Prophylactic antibiotics, post partum oxytocics and the use of misoprostol post operatively helped to prevent further post partum hemorrhage.

When a patient isinitially opted for conservative management, the possibility of recurrence should be discussed [8]. Furthermore, placentation should be carefully monitored for recurrence in any subsequent pregnancy, particularly if the placenta is located at the same site as the previous placenta accreta.

Conservative treatment for placenta accreta may be an alternative procedure in some selected cases.

## Conclusion

We suggest an alternative approach for managing uterine inversion caused by placenta accreta that involved conservative management. This way hysterectomy was avoided and fertility was preserved.

## Abbreviations

CS: Caesarean Section; FFP: Fresh Frozen Plasma

## Consent

Written informed consent was obtained from the patient for publication of this case report and accompanying images. A copy of the written consent is available for review by the Editor – in Chief of this journal.

## Competing interests

The authors declare that they have no competing interests.

## Authors' contributions

All authors have made substantial contribution to concept this case report.
